# TMSOTf assisted synthesis of 2’-deoxy-2’-[^18^F]fluoro-β-D-arabinofuranosylcytosine ([^18^F]FAC)

**DOI:** 10.1371/journal.pone.0196784

**Published:** 2018-05-01

**Authors:** Kishore K. Gangangari, John L. Humm, Steven M. Larson, Naga Vara Kishore Pillarsetty

**Affiliations:** 1 Department of Radiology, Memorial Sloan Kettering Cancer Center, New York, NY, United States of America; 2 Department of Chemistry, Hunter College and PhD Program in Chemistry, The Graduate Center of the City University of New York, New York, NY, United States of America; 3 Department of Medical Physics, Memorial Sloan Kettering Cancer Center, New York, NY, United States of America; 4 Molecular Pharmacology Program, Memorial Sloan Kettering Cancer Center, New York, NY, United States of America; 5 Department of Radiology, Weill Cornell Medical College, New York, NY, United States of America; Brandeis University, UNITED STATES

## Abstract

[^18^F]FAC (2’-deoxy-2’-[^18^F]fluoro-β-D-arabinofuranosylcytosine, **1**) is a versatile probe for imaging deoxycytidine kinase (dCK) expression levels *in vivo*. dCK is responsible for phosphorylation of deoxycytidine (dC, **2**) and other nucleoside analogs, plays a key role in immune activation and has demonstrated to be one of the key enzymes in activating nucleoside based drugs including gemcitabine. Reported synthesis of [^18^F]FAC is high yielding but is quite challenging requiring bromination using HBr and careful drying of excess HBr which is critical for successful synthesis. Here in we report a simplified trimethylsilyl trifluoromethanesulfonate (TMSOTf) assisted synthesis of [^18^F]FAC eliminating the need of bromination and drying. [^18^F]FAC (β-anomer) was synthesized with average isolated decay corrected yield of 10.59 + 4.2% (n = 6) with radiochemical purity of >98% and total synthesis time of 158 + 19 min.

## Introduction

**Fig 1:**
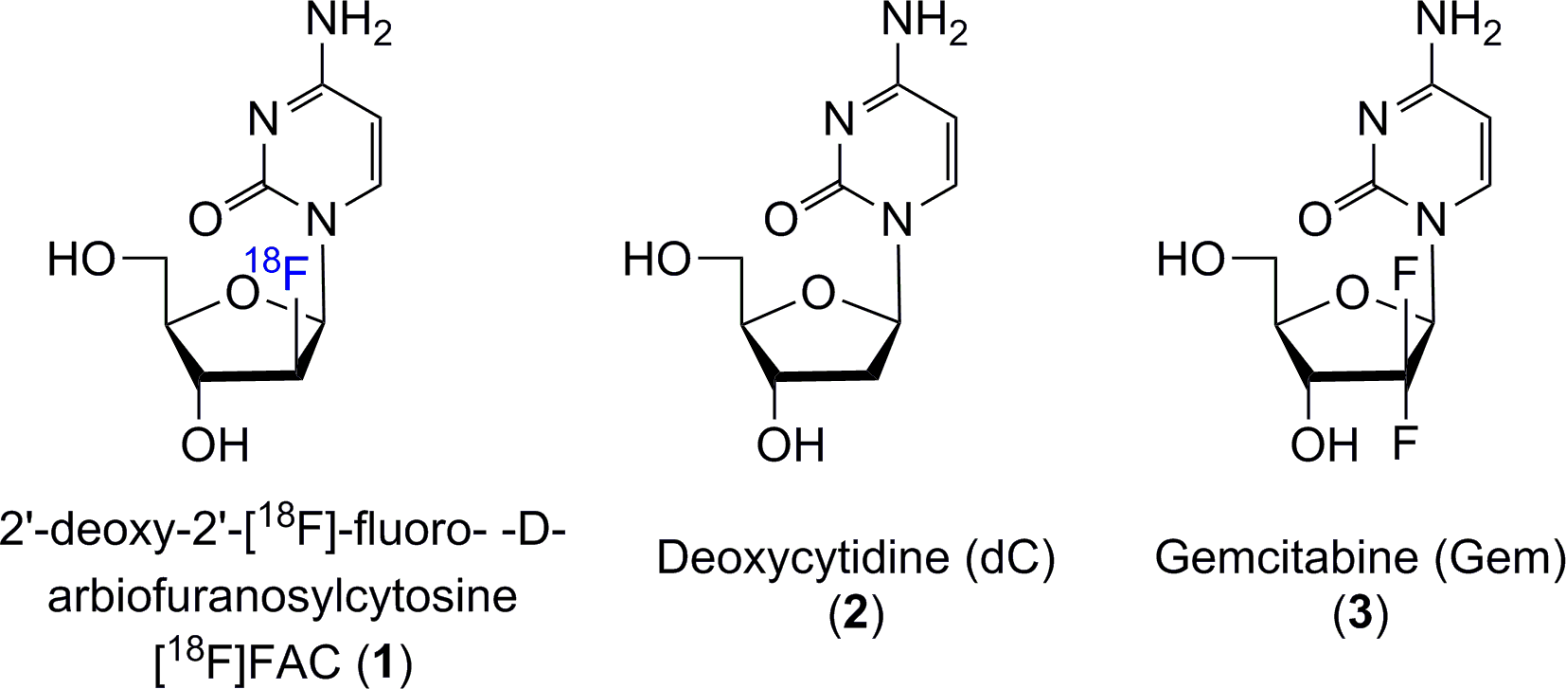
Chemical structures of 2’-deoxy-2’-[^18^F]fluoro-β-D-arbiofuranosylcytosine ([^18^F]FAC) and its analogs.

[^18^F]FAC (1-(2’-deoxy-2’-[^18^F]fluoro-β-D-(arabinofuranosyl)cytosine), **1**) (Fig 1) is a close analog of deoxycytidine (dC, **2**) and is an efficient substrate for phosphorylation by deoxycytidine kinase (dCK). It was developed by Radu’s group at UCLA for imaging lymphoid organs and immune activation because of critical role played by dCK in these processes.[1, 2] Being a critical enzyme, dCK is expressed constitutively in all cells at a low background level but significantly increased expression is observed in lymphoid cells and many cancer cells.[3] In addition to phosphorylation of dC, dCK catalyzes phosphorylation of other nucleosides such as deoxyadenosine, deoxyguanosine.[4] This property has been utilized in development of several nucleoside based prodrugs used in cancer chemotherapy including gemcitabine (Gem, **3**). Gem is first line of treatment for pancreatic patients and other solid tumors and has a very low response rate (5–30%).[5] The low level of response can be partly attributed to low levels of dCK in the tumors [6]. Thus [^18^F]FAC can act as a PET tracer to non-invasively image the levels of dCK in cancer patients. We have previously demonstrated that [^18^F]FAC can also act as a surrogate marker for gemcitabine.[7]

**Fig 2:**
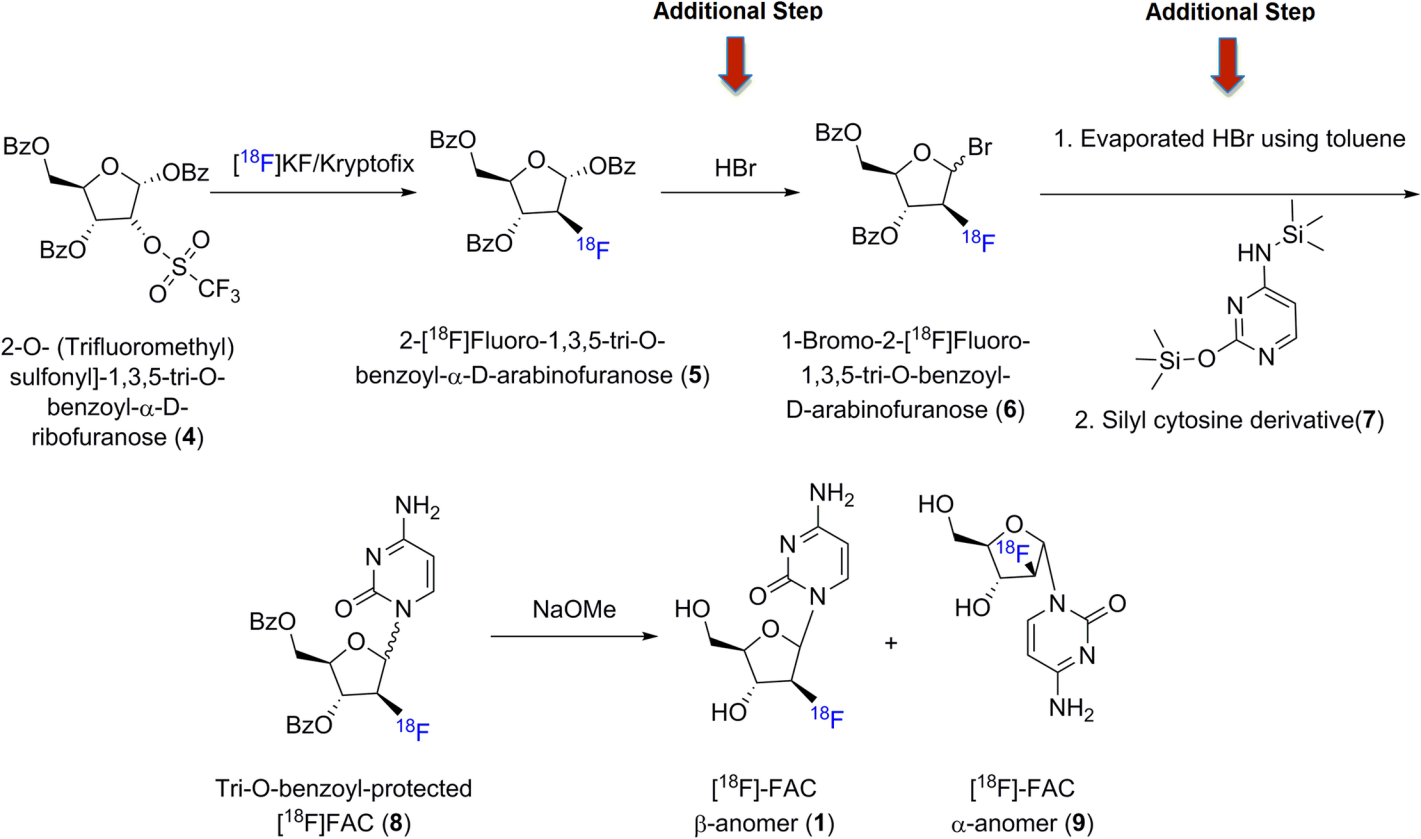
Classical synthesis of [^18^F]FAC employing HBr for activation and coupling to the cytosine silylether developed by Radu’s group.

Previous synthesis of [^18^F]FAC (Fig 2) was based on the original synthesis of cold fluorinated analog FAC by Wright and Fox’s well known nucleophillic substitution method.[8] This multistep synthesis has been adapted to synthesis of various fluorine-18 labeled nucleosides including 2′-deoxy-2′-fluoro-5-iodo-1-*β*-D-arabinofuranosylcytosine ([^18^F]FIAC) [9] and other uridine derivatives.[10] Briefly, [^18^F]FAC was synthesized starting from flourine-18 labeling of commercially available precursor **4** followed by activation of C-1 of sugar with bromine using HBr in acetic acid(HOAc) to facilitate condensation with cytosine silyl derivative. Excess of HBr is evaporated using toluene before adding **7** followed by deprotection and HPLC purification of alpha and beta anomers. This method provides moderate yields of [^18^F]FAC in our experience and has been successfully automated by other groups using Elixys® radiosynthesizer with excellent yields.[11] In an effort to reduce the complexity of the synthesis, a one step late stage fluorination of [^18^F]FAC was developed by Meyer et. al. [12] with comparable total synthesis time and moderate yield of 4.3–5.5% (d.c). This method presents advantages of elimination of many intermediate steps, but the precursor synthesis is challenging and is not commercially available. Therefore we explored methods to reduce the complexity of the synthesis of [^18^F]FAC.

We have previously published trimethylsilyltriflate (TMSOTf)-assisted methodology based on Vorburggen’s trimethylsilyl triflate[13] assisted coupling to synthesize fluorine-18 labeled 2′-deoxy-2′-fluoro-5-iodo-1-β-D-arabinofuranosyluracil (FIAU) and other 5 substituted uracil derivatives to give alpha and beta anomers of 5-substituted uridine derivatives directly from the 1-benzoyl sugar (**5**) instead of corresponding 1-bromo-derivative(**6**).[14–16] This method using TMSOTf or strong Lewis acid such as SnCl_4_ as a catalyst completely reduced the overall synthesis by 2 steps—bromination and evaporation of excess HBr.

Here in we report an alternative trimethylsilyl trifluoromethanesulfonate (TMSOTf) assisted three step synthesis of [^18^F]FAC that eliminates the need for bromination and drying steps with comparable time and yields for total synthesis. The reduction in number of steps, purifications and reaction times makes this method amenable to manual synthesis in one pot.

## Materials and methods

All reagents and solvents were obtained from Sigma-Aldrich or Thermo Fisher Scientific and used without further purification. No-carrier added [^18^F]fluoride in water was obtained from the Radioisotopes and Molecular Imaging Probes core facility at MSKCC. FAC was purchased from Carbosynth, Berkshire, UK (CAS: 56632-83-8, Product code: ND08343). 2-(O)-(trifluoromethylsulfonyl)-1,3,5-tri-O-benzoyl-α-D-arabinofuranose **(4)** was purchased from Carbosynth, Berkshire, UK (CAS: 97614-41-0, Product code: MT07900). Sep-Pak Accell plus QMA plus Short Cartridge, 360 mg Sorbent per Cartridge, 37–55 μm Particle Size, (WAT020545) were purchased from Waters (MA, USA). C18-mini column (Strata C18-E (55 μm, 70 Å) 50mg/1mL) [8B-S001-EAK] were purchased from Phenomenex®, CA. HPLC purification and analysis were performed on a Shimadzu HPLC system equipped with a binary high pressure gradient solvent delivery module LC 10A and SPD-20A UV dual wavelength detector connected to a bioscan flow-count radio-HPLC detector system for gamma detection. Crude products were purified and analyzed for purity on reversed phase C18 column using Waters XBridge™ 5 μm, 10 x 250 mm [186008167] (for preparative scale) and Phenomenex® Gemini 5 μm, 250 x 4.6 mm 110 Å [00G-4435-E0], (for analytical HPLC). Purification was carried out using an isocratic solvent system of 2% acetonitrile in 0.1% trifluoroacetic acid at a flow rate of 8 mL/min. Analysis of the purified product was carried out using 3% acetonitrile in 0.1% trifluoroacetic acid at a flow rate of 1 mL/min. Microwave systems used for the reactions were obtained from Biotage Inc (Initiator 2.5, Charlotte, NC) and microwave was not used unless explicitly mentioned. The yields are reported as average ± standard deviation. The yields reported are isolated yields from 6 independent experiments and decay corrected to the time at the elution of fluorine-18 radioactivity from the QMA cartridge.

### Synthesis of [^18^F]FAC (2'-deoxy-2'-[^18^F]fluoro-β-D-(arabinofuranosyl)cytosine) (1)

**Fig 3:**
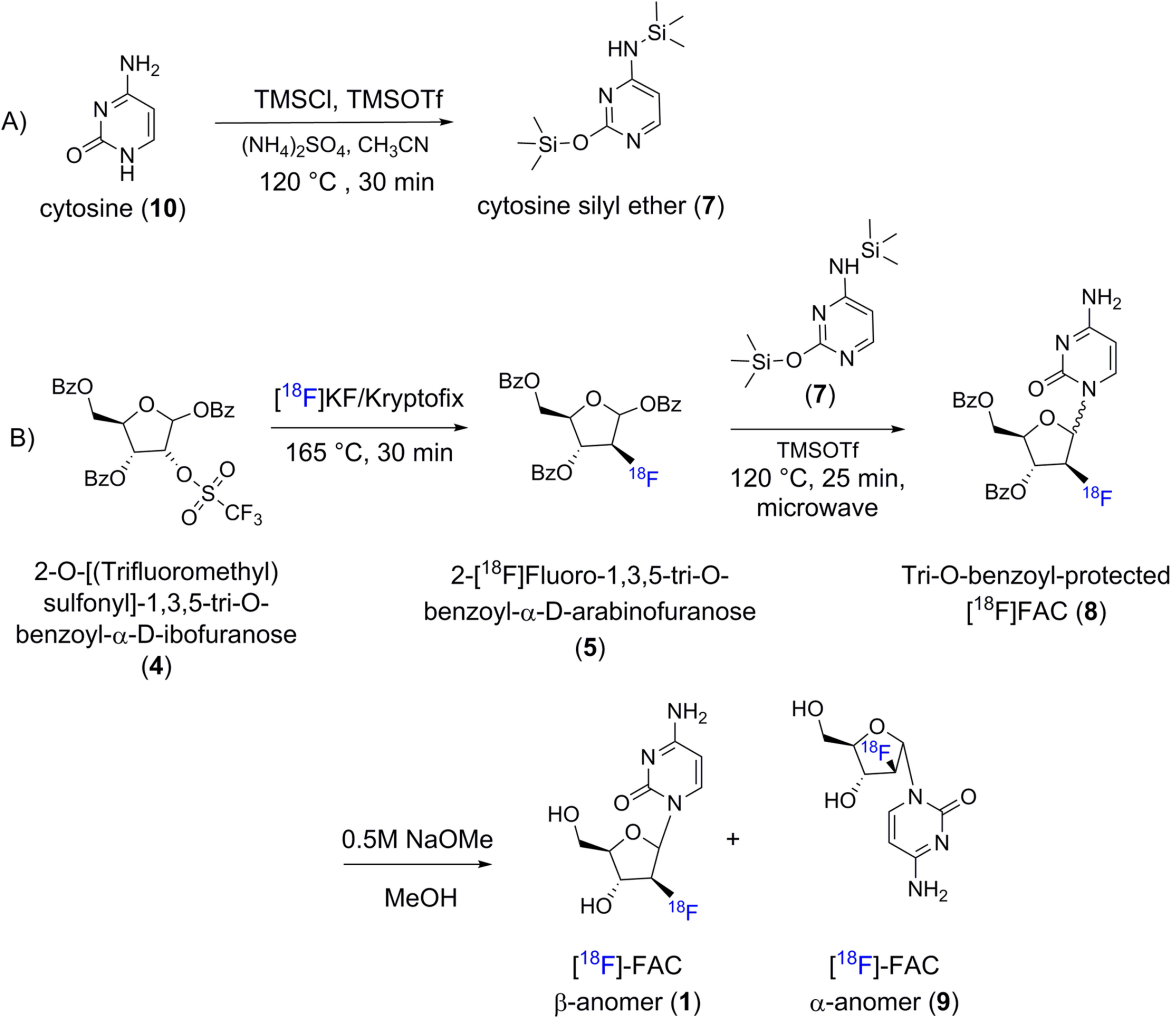
Synthetic scheme for the synthesis of [^18^F]FAC using TMSOTf assisted coupling of 1.3.5-tribenzoyl-2-deoxy-2-[^18^F]fluoro-arabinofuranose with cytosine silyl ether using microwave heating.

Overall synthetic strategy is given in Fig 3**.** The synthetic approach was based on our earlier method for synthesis of FIAU and its analogs originally developed by Vorbruggen and optimized for pyrimidine nucleosides. [17] Synthetic scheme consists of four major steps as described below.

1. **Synthesis of cytosine silyl ether (N-(trimethylsilyl)-2-((trimethylsilyl)oxy)pyrimidin-4-amine, 7):** Cytosine silyl ether **(7)** was synthesized by heating a mixture of cytosine **(10)** 12 mg (0.11 mmoles), TMSOTf (100 μL, 123 mg, 0.55 mmoles), and 1,1,1-Trimethyl-N-(trimethylsilyl)silanamine (HMDS, 100 μL, 0.47 mmoles) in acetonitrile (300 μL) at 120°C for 20 min in a microwave vial. The product was used for coupling reaction, without any further purification.
2. **Synthesis of 2-deoxy-2-[**^**18**^**F]fluoro-1,3,5-tri-O-benzoyl-**α**-D-arabinofuranose (5) (Fluorination of 2-O-[(Trifluoromethyl)sulfonyl]-1,3,5-tri-O-benzoyl-α-D-ribofuranose (4)):** ^18^F in the form of [^18^F]HF, was loaded onto QMA cartridge preconditioned by passing 5 mL of 0.25 M KHCO_3_ followed by 20 mL of deionized water and eluted with 1 mL of 90% acetonitrile containing KHCO_3_ (1.6 mg, 15.9 μmol) and kyrptofix (10 mg, 26.4 μmol) into a 10 mL reacti-vial™. The water acetonitrile azeotrope was removed by heating the vial to 105–110°C under a slow stream of argon gas (150–175 mL/min). To the dried reacti-vial™, an additional 0.7 mL of anhydrous acetonitrile was added and the solvent was removed as described above and the whole process was repeated 2 additional times. The reacti-vial™ was cooled to room temperature (RT) and radioactivity was extracted using anhydrous acetonitrile (0.4 mL) and added to 2-O-(trifluoromethylsulfonyl)-1,3,5-tri-O-benzoyl-α-D-arabinofuranose **(4)** (15 mg) in a sealed microwave vial and the reaction mixture was heated at 120°C for 30 minutes. The product was used for reactions without any further purification.
3. **Synthesis of 3’,5’-O-dibenzoyl-(2-[**^**18**^**F]fluoro-**β**-D-(arabinofuranosyl) cytosine (8) (Coupling of cytosine silyl ether (7) and 2-deoxy-2-[**^**18**^**F]fluoro-1,3,5-tri-O-benzoyl-**α**-D-arabinofuranose(5)):** The reaction mixture was cooled to RT and added to vial containing silyl ether **(7)** solution and TMSOTf (100 μL) and HMDS (100 μL) in acetonitrile (300 μL) and the reaction mixture was heated to 120°C for 25 min using microwave reactor under sealed conditions. Then reaction mixture was cooled to RT and passed through a silica Sep-Pak^®^ plus column (pre-conditioned with 5 mL of hexane) and eluted into a 10 mL reacti-vial™ with 10% MeOH in CH_2_Cl_2_ (2 x 1.25 ml). The solvents were removed by heating the reacti-vial™ under argon flow at 100 °C and used for the next step directly.
4. **Deprotection of 3’,5’-O-dibenzoyl-(2-[**^**18**^**F]fluoro-**β**-D-(arabinofuranosyl) cytosine (8):** To the vial containing **8**, 0.5 mL of 4.6 M sodium methoxide in MeOH (25% w/v) was added and the reaction mixture was heated at 80 °C for 10 min for deprotection of the benzoyl groups. The reaction mixture was treated with glacial acetic acid (120 μL) and the solvent was removed under argon stream at 80°C. The residue was formulated in 1% acetonitrile and passed through C18-mini column (Strata C18-E (55 μm, 70 Å) 50mg/1mL) to remove insoluble impurities. The crude product was purified using reversed phased HPLC (on the preparative column) using an isocratic solvent system of 1% acetonitrile in 0.1% trifluoroacetic acid in water. The radioactive fraction corresponding to the product peak [^18^F]FAC **(9,** β-anomer) was collected and solvent evaporated under reduced pressure. The identity of the product [^18^F]FAC **(9)** was verified by co-injecting with commercially available non-radioactive analog on an HPLC analytical column using an isocratic solvent system of 3% acetonitrile in 0.1% trifluoroacetic acid in water.

## Results

[^18^F]FAC was synthesized in yields (d.c.) ranging from 2.2–11.2% with average yield of 5.95 + 1.6% with radiochemical purity of >95%. The molar activity of [^18^F]FAC was found to be 125–700 mCi/μmol (13–26 GBq/μmole) and α- to β-anomer ratio of about 2:1. We did not attempt to improve specific activity of the product as the uptake of FAC is not influenced in given specific activity ranges. Manual synthesis was accomplished in 158 + 19 min from obtaining flourine-18 in water.

## Discussion

An alternative method for synthesis of [^18^F]FAC (**1**) was developed that reduces the number of steps of conventional synthesis by 2 with comparable yields and synthesis times. Lewis acid TMSOTf was utilized for direct coupling of un-activated deoxysugar with cytosine.

The fluorination of 2-O-(trifluoromethylsulfonyl)-1,3,5-tri-O-benzoyl-α-D-arabinofuranose (**4**) has been well established and therefore no attempts to optimize the reaction were attempted. The crude fluorinated product was used directly without any Sep-Pak® purification and therefore saving about 10–15 min of time for the step.

Coupling of 1,3,5-tribenzoyl-2-deoxy-2-[^18^F]fluoro-arabinofuranose (**5**) with cytosine silyl ether (**7**) was achieved using 100 μL of trimethylsilyl triflate in acetonitrile. This resulted in an efficient coupling reaction and [^18^F]FAC was obtained after deprotection. The coupling was performed in acetonitrile as solvent as this gave more consistent yields and easier evaporation post Sep-Pak® purification albeit with higher α-anomeric product. Due to usage of polar CH_3_CN for the coupling reaction, the undesired α-anomer was a major product with α- to β-anomer ratio of about 2:1. [18] As demonstrated by Alauddin et al, [19] the anomeric ratios are highly dependent on the polarity of the solvent used for coupling step with non-polar solvents favoring β-anomer over α-anomer.

Microwave heating was employed as it gave consistent results. Heating the coupling reaction on a heating block also provided us with the product, albeit in lower yields (<1% d.c.). However we did not attempt to optimize coupling reaction with conventional heating.

This method offers an advantage of employing freshly synthesized cytosine silyl ether (which also employs TMSOTf for protection) without further purification in the synthesis of [^18^F]FAC, which in our hands showed considerable increase in the product yields. It was observed that the product yields were the highest when TMSOTf was used freshly after opening the vial and the coupling and overall yields declined with storage. This could be attributed to the high reactivity of TMSOTf that results in hydrolysis of TMSOTf even when all precautions were observed.

**Fig 4:**
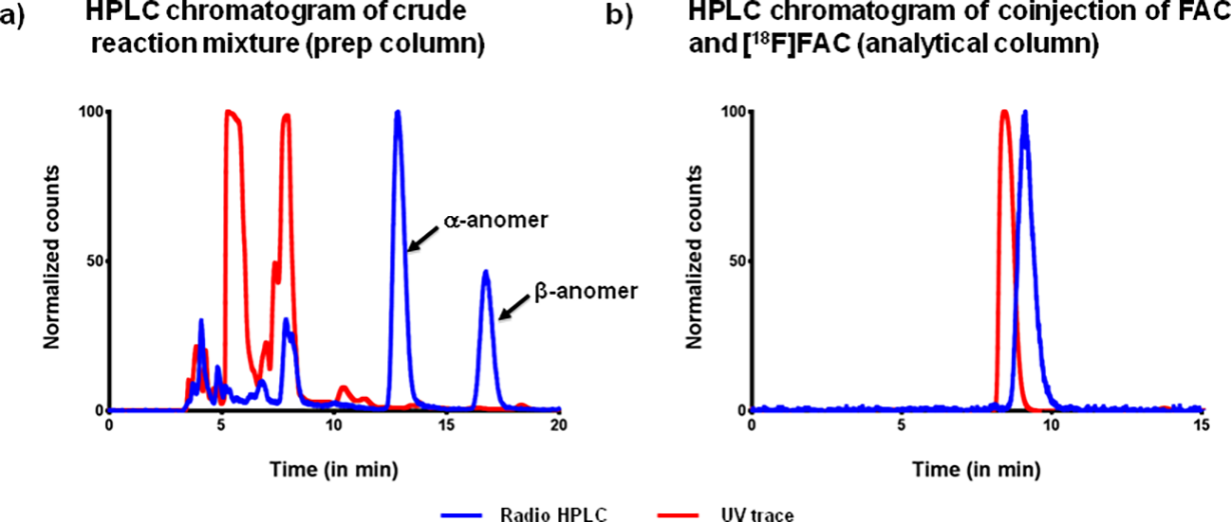
a) HPLC chromatogram showing the purification of [^18^F]FAC using a semi preparative HPLC system. Blue line: radioactive signal from radioactive detector Red line: UV signal from UV detector @ 254 nm. b) Quality control analysis of [^18^F]FAC with analytical HPLC system showing coinjection of purified [^18^F]FAC from preparatory column and non-radioactive FAC.

The HPLC purification of crude product containing a mixture of alpha and beta anomers (α:β = 2:1) was easily accompanied using regular C-18 RP column (250 x 10 mm). Under the given HPLC conditions (1% acetonitrile in water, 0.1%TFA), [^18^F]FAC eluted with a retention time of ~17 min preceded by alpha anomer at ~13.5 minutes (Fig 4). For the HPLC purification it is important to ensure that the crude product is completely free of polar acetonitrile and methanol and reformulated in 1% acetonitrile in water. Presence of polar solvent can result in elution of product in dead volume and therefore has to be minimized. Ensuring the evaporation of polar solvents results in a clean separation of the free fluoride, free sugar, and the α-anomer from the product. The crude compound was purified and coinjected with cold FAC on the HPLC to confirm the identity of the product.

While TMSOTf assisted synthesis [^18^F]FAC reduces the synthesis time and purification steps, it has its own drawbacks. It has to be noted that the hydrolysis product triflic acid could be as corrosive as HBr/AcOH and bromine vapors, products of traditional synthesis. Moreover TMSOTf is highly reactive and fumes violently in presence of moisture posing handling issues to untrained personnel. As mentioned earlier, the yield of the final product decreased with storage. Additionally, the coupling reaction is efficient only using the microwave as very poor yields resulted using the conventional heating methods.

To summarize, the current method that utilizes TMSOTf assisted synthesis of [^18^F]FAC reduces the number of steps while providing sufficient yields for carrying out *in vivo* studies. The reduction in number of steps is a big advantage for laboratories that lack automated synthesizers and where manual synthesis is routinely employed.

## Conclusions

An alternative shorter method for synthesis of [^18^F]FAC is developed. Overall synthesis was shortened by eliminating the bromination and evaporation steps and by employing microwave heating for the coupling of deoxysugar with the cytosine. This method provides reliably reproducible yields and is easily amenable for manual synthesis.
